# Comparison of peri-implant submucosal microbiota in arches with zirconia or titanium implant-supported fixed complete dental prostheses: a study protocol for a randomized controlled trial

**DOI:** 10.1186/s13063-020-04853-7

**Published:** 2020-11-27

**Authors:** Pingyi Jia, Jingwen Yang, Zhaoguo Yue, Jianzhang Liu, Qi Liu, Zhongning Liu, Lin Tang, Jianxia Hou

**Affiliations:** 1 Department of the Fourth Clinical Division, Peking University School and Hospital of Stomatology; National Engineering Laboratory for Digital and Material Technology of Stomatology; Research Center of Engineering and Technology for Digital Dentistry of Ministry of Health; Beijing Key Laboratory of Digital Stomatology, Beijing, People’s Republic of China; 2 Department of Prosthodontics, Peking University School and Hospital of Stomatology; National Engineering Laboratory for Digital and Material Technology of Stomatology; Research Center of Engineering and Technology for Digital Dentistry of Ministry of Health; Beijing Key Laboratory of Digital Stomatology, Beijing, People’s Republic of China; 3 Department of Periodontology, Peking University School and Hospital of Stomatology; National Engineering Laboratory for Digital and Material Technology of Stomatology; Research Center of Engineering and Technology for Digital Dentistry of Ministry of Health; Beijing Key Laboratory of Digital Stomatology, No. 22, Zhongguancun Avenue South, Haidian District, Beijing, 100081 People’s Republic of China; 4 BYBO Dental Hospital, Qinian Street, Dongcheng District, Beijing, 100062 People’s Republic of China

**Keywords:** Dental implants, Edentulous, Microbiota, Bio-complication, Zirconia, Titanium

## Abstract

**Background:**

The success rate of implant-supported prostheses for edentulous patients is relatively high. However, the incidence of biological complications, especially peri-implant mucositis and peri-implantitis, increases yearly after the placement of prostheses. The accumulation of pathogenic bacteria adjacent to a prosthesis is the main cause of biological complications. Titanium, one of the classical materials for implant-supported prostheses, performs well in terms of biocompatibility and ease of maintenance, but is still susceptible to biofilm formation. Zirconia, which has emerged as an appealing substitute, not only has comparable properties, but presents different surface properties that influence the adherence of oral bacteria. However, evidence of a direct effect on oral flora is limited. Therefore, the aim of the present study was to assess the effects of material properties on biofilm formation and composition.

**Methods:**

The proposed study is designed as a 5-year randomized controlled trial. We plan to enroll 44 edentulous (mandible) patients seeking full-arch, fixed, implant-supported prostheses. The participants will be randomly allocated to one of two groups: group 1, in which the participants will receive zirconia frameworks with ceramic veneering, or group 2, in which the participants will receive titanium frameworks with acrylic resin veneering. Ten follow-up examinations will be completed by the end of this 5-year trial. Mucosal conditions around the implants will be recorded every 6 months after restoration. Peri-implant submucosal plaque will be collected at each reexamination, and bacteria flora analysis will be performed with 16S rRNA gene sequencing technology in order to compare differences in microbial diversity between groups. One week before each visit, periodontal maintenance will be arranged. Each participant will receive an X-ray examination every 12 months as a key index to evaluate the marginal bone level around the implants.

**Discussion:**

The current study aims to explore the oral microbiology of patients following dental restoration with zirconia ceramic frameworks or titanium frameworks. The features of the microbiota and the mucosal condition around the two different materials will be evaluated and compared to determine whether zirconia is an appropriate material for fixed implant-supported prostheses for edentulous patients.

**Trial registration:**

International Clinical Trials Registry Platform (ICTRP) ChiCTR2000029470. Registered on 2 February 2020. http://www.chictr.org.cn/searchproj.aspx?

## Background

Full-arch fixed implant-supported prostheses have achieved satisfactory clinical results in edentulous patients with long-term implant survival of almost 100% [1]. However, biological complications after the placement of implant-supported fixed complete dental prostheses (IFCDPs) occur continuously over time [2]. Previous studies have indicated that peri-implant mucositis is associated with plaque accumulation [3, 4] around implants and prostheses and that the hygiene of IFCDPs could be influenced by the distance between the inserted implants in the jaw, the palatal extension of the prostheses [5], and the implant materials [6]. Investigations on the bacterial adhesion on titanium, the most commonly used materials in daily practice, have revealed that the corrosion of titanium increases plaque accumulation [7]. Zirconia ceramic frameworks, with or without ceramic veneering, is considered a promising material with good bio-compatibility and mechanical properties [8]. Therefore, many studies are currently investigating bacterial adhesion on zirconia discs/abutments as compared with titanium [9–12]. Results of in vitro studies suggest that zirconia is more resistant to bacterial colonization. However, in vivo evidence of bacterial control around implants remains controversial. For example, Grossner-Schreiber et al. [13] found that bacterial counts were higher with titanium discs than zirconia, while Scarano et al. [14] showed that bacterial adhesion was significantly higher with pure titanium surfaces as compared with zirconium oxide surfaces and Egawa et al. [15] found that bacterial adherence to titanium was comparable to that of zirconia if the surfaces of both materials had mirror-like flat textures.

These discrepancies may be due to differences in study design. First, material surface roughness is a key factor for bacterial colonization [16, 17]. Therefore, statistical analyses of bacterial adhesion on materials without uniform surface roughness are not comparable [9–11, 14]. Second, studies that included patients with partial edentulism could not exclude the influence of the oral flora on the remaining teeth on the establishment of peri-implant microbiota, which might have influenced comparisons of peri-implant microbiota between different groups [12–14]. To eliminate such interfering factors, full-arch restoration with materials with equal smoothness should be investigated. A previous study of 20 edentulous investigated early material colonization around zirconia vs. titanium abutments before final restorations found no difference in early bacterial colonization between the two materials. Since this observation period was relatively short (3 months) [10], additional studies are needed to determine whether there are differences in the performance of frameworks after final restoration. Considering that bacterial colonization might be influenced by aging of zirconia [18] and long-term corrosion of titanium [7], a comparative study with a longer observation period is warranted.

Therefore, there is a need for a randomized controlled study with a sufficient sample size and long-term observation period to provide direct clinical evidence about the effect of the surface properties of full-arch implant-based prostheses on submucosal microbiota. The aim of the current study is to compare the clinical conditions and peri-implant microbiota of edentulous patients following dental restoration with titanium-based vs. zirconia-based prostheses.

## Objectives

The aims of the study are (1) to investigate and compare the implant survival and biological complication rates between two different restorative materials on full-arch fixed implant-supported rehabilitation and (2) to compare the effect of two different restorative materials on the diversity of microbiota colonizing full-arch fixed implant-supported restorations (i.e., zirconia frameworks with ceramic veneering vs. titanium frameworks with acrylic resin veneering).

## Methods and study design

### Study design and setting

**Fig. 1:**
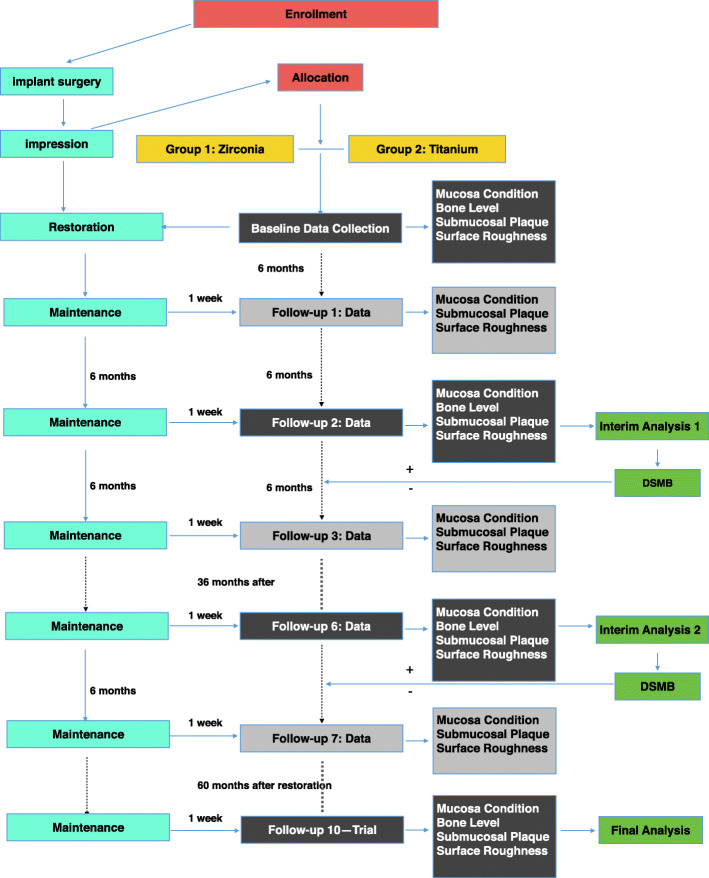
Consolidated Standards of Reporting Trials diagram

**Fig. 2:**
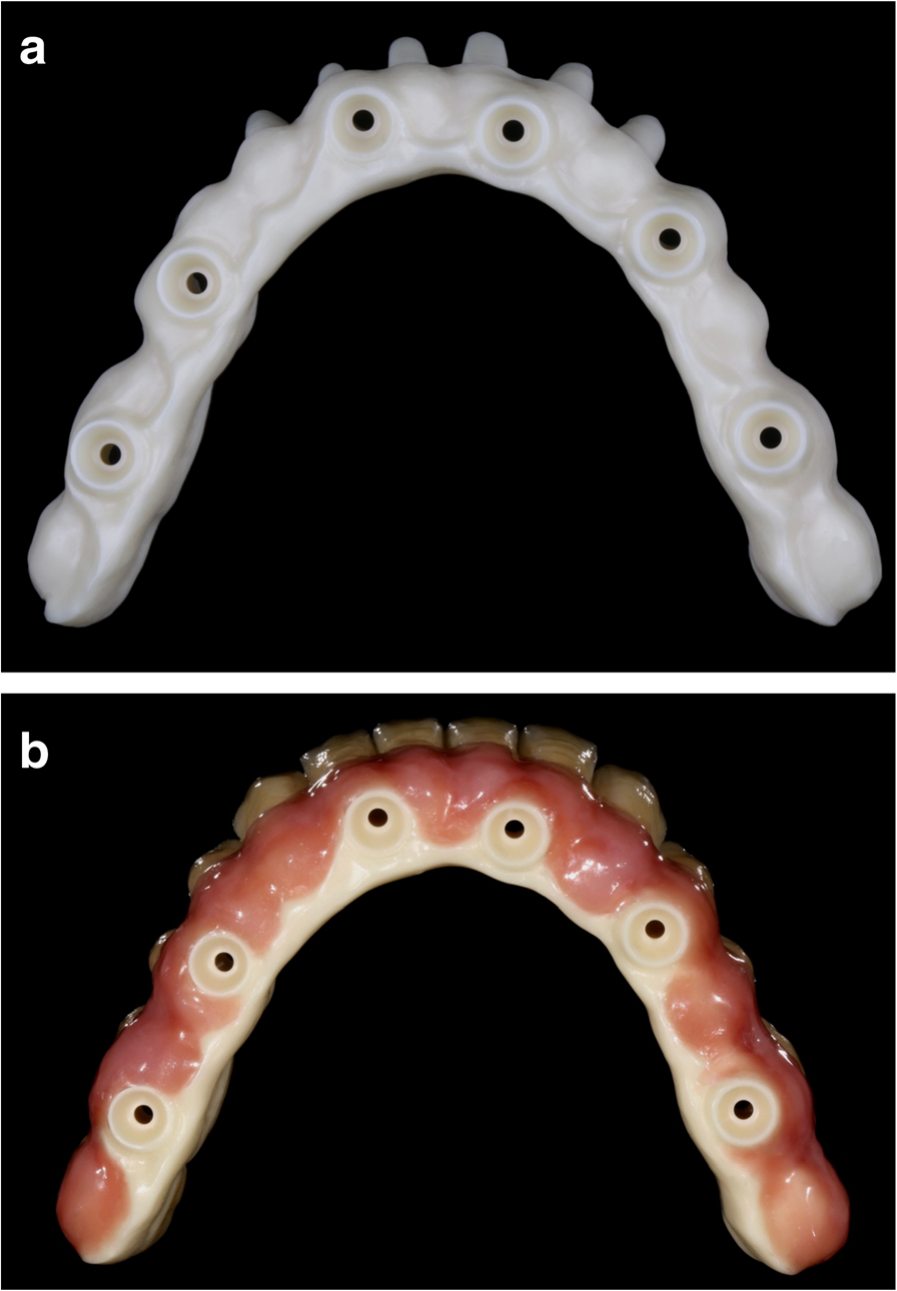
Zirconia framework with ceramic veneering (group 1). **a** Zirconia framework. **b** Zirconia framework with ceramic veneering

**Fig. 3:**
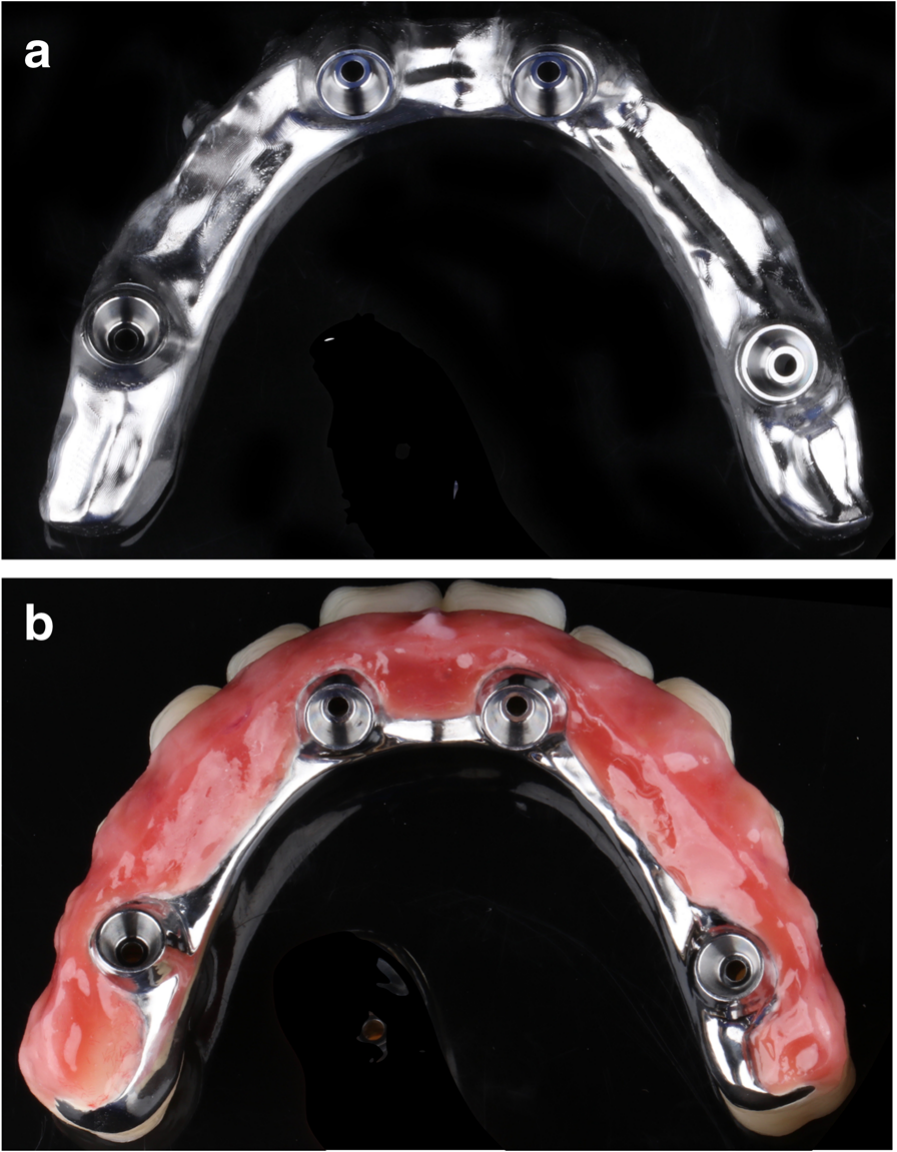
Titanium framework with acrylic resin veneering (group 2). **a** Titanium framework. **b** Titanium framework with acrylic resin veneering

The proposed study is designed as a pragmatic randomized controlled trial with a 5-year follow-up period. A Consolidated Standards of Reporting Trials diagram is presented in Fig. 1. We plan to enroll 44 edentulous patients (mandible) in need of dental implant-based prostheses. The goal of the treatment protocol is to rehabilitate these patients with full-arch fixed implant-supported prostheses with placement of 4–6 titanium implants in the mandible. The patients will be randomly assigned to receive restorative materials of zirconia frameworks with ceramic veneering (Fig. 2) or titanium frameworks with acrylic resin veneering (Fig. 3). Both groups will receive titanium implants with sandblasted, large grid, and acid-etched surfaces. The trial will last for 5 years, and the patients will be followed up at 6-month intervals. During each visit, the patient will undergo a clinical examination, surface roughness test, and submucosa plaque collection, while biological and mechanical complications will be treated. One week before each visit, the patient will receive periodontal maintenance, including ultrasonic debridement using an ultrasonic device with polyetheretherketone-coated tips (AIRFLOW® Prophylaxis Master; EMS Dental, Nyon, Switzerland) combined with erythritol air-polishing (AIR-FLOW® handy 3.0 PERIO; EMS Dental). X-ray assessment will be performed yearly. A treatment timeline is shown in Table 1.

**Table 1:** Participant timeline

TIMEPOINT	T0	T1	T2	Month0	1 week before Month 6	Month6	1 week before Month 12	Month12	1 week before Month 18	Month18	1 week before Month 24	Month24	1 week before Month 30	Month30	1 week before Month 36	Month36	1 week before Month 42	Month 42	1 week before Month 48	Month 48	1 week before Month 54	Month 54	1 week before Month 60	Month 60
**ENROLMENT:**																								
**Eligibility screen**	X																							
**Informed consent**	X																							
**Implant surgery**		X																						
**Impression**			X																					
**Allocation**				X																				
**INTERVENTIONS:**																								
**Final restoration:** **titanium**				X																				
**Final restoration:** **zirconia**				X																				
**MAINTANENCES:** **Ultrasonic debridement with PEEK tips**					X		X		X		X		X		X		X		X		X		X	
**ASSESSMENTS:**																								
**Implant survival** **rate**								X								X								X
**Peri-implant plaque index**				X		X		X		X		X		X		X		X		X		X		X
**Bleeding on probing**				X		X		X		X		X		X		X		X		X		X		X
**Suppuration**				X		X		X		X		X		X		X		X		X		X		X
**Probing depth**				X		X		X		X		X		X		X		X		X		X		X
**Mechanical complication**						X		X		X		X		X		X		X		X		X		X
**Paralleling X-ray**				X				X				X				X				X				X
**Microbiota sample collection**				X		X		X		X		X		X		X		X		X		X		X
**Surface roughness**				X		X		X		X		X		X		X		X		X		X		X

The primary outcomes of the present study are the implant survival rate, peri-implant plaque index (PLI), peri-implant mucosal status, marginal bone resorption rate, and diversity of peri-implant submucosal bacteria species, while the secondary outcomes are the mechanical complication rate and surface roughness measurement.

### Ethical considerations

The proposed study is designed as a prospective, single-center, randomized controlled trial. The study protocol was approved by the Ethics Committee of the Stomatology School and Hospital of Peking University (Beijing, China; approval no. PKUSSIRB-202054027). In addition, the study has been registered with the ClinicalTrials.gov website (https://clinicaltrials.gov/; identifier no. ChiCTR2000029470).

The systematic health status of all study participants will be recorded. Before implant surgery, all patients will be instructed on how to maintain good oral hygiene and undergo clinical and radiographic assessments.

### Recruitment

Patient recruitment was initiated in May 2020. Approximately 50 edentulous patients receive full-arch implant-supported prosthesis every year in this hospital. Announcements were posted in the registration and waiting room areas, and it is expected that 36 eligible participants will be recruited within 2 years. Eligible patients will receive the study protocol and a consent form, which must be completed prior to study inclusion.

Inclusion criteria

1. Edentulous jaw
2. American Society of Anesthesiologists physical status classification of I or II
3. Age > 22 years
4. Good oral hygiene and good compliance
5. Signing of an informed written consent form

Exclusion criteria

1. Poor oral hygiene and uncontrolled peridontitis
2. Antibiotic use within the past 3 months
3. Systemic disease: uncontrolled diabetes mellitus, cardiovascular disease, immunodeficiency disorders, blood disorders such as coagulation disorders, severe osteoporosis, and any metabolic disease that might influence the rate of bone resorption
4. Long-term medication use: steroid, anti-epileptic drugs, drugs favoring gingival overgrowth, bisphosphonates (bisphosphonate injections or more than 4 years of oral bisphosphonate use), and any medication that influences the rate of bone loss or survival of dental implants, such as selective serotonin re-uptake inhibitors, proton-pump inhibitors, or beta-blockers for the treatment of hypertension
5. Infection of human immunodeficiency virus, hepatitis B virus, or syphilis
6. Bruxism
7. Uncontrolled infection in the area intended for implant placement or other areas
8. Maxillofacial tumor
9. Face–neck radiotherapy
10. Mental illness or high expectations
11. Unable to sign the informed consent form
12. Smokers: use of cigarettes, electronic cigarettes, or water pipes

### Interventions

The aim of the treatment protocol is to rehabilitate these patients with full-arch fixed implant-supported prosthesis with placement of 4–6 implants in the mandible. Patients will be randomly assigned to receive one of two restorative materials: zirconia ceramic frameworks with or without ceramic veneering vs. titanium frameworks with acrylic resin veneering. Before prosthesis fitting, the patients will undergo a standard polishing procedure (GB/T 6060.2-2006) and examination to ensure the same surface roughness between groups. The surface roughness of all specimens will be measured with the use of a profilometer (Mitutoyo Surftest SJ-401; Mitutoyo Corporation, Kawasaki, Japan), as described in a previous study [19]. The implant number, implant site, implant diameter, implant length, implant torque, distances between the inserted implants, and shapes of the intaglio surface will be recorded as basic information.

### Primary and secondary outcome variables

The primary outcomes are the implant survival rate, PLI, peri-implant mucosal conditions, marginal bone resorption, and diversity of peri-implant submucosal bacteria species, while the secondary outcomes are the mechanical complication rate and surface roughness of the two treatment groups.

### Clinical assessment

Clinical examinations will be performed at baseline (immediately after prosthesis placement) and every 6 months after implantation of the final prosthesis.

The following parameters will be evaluated: PLI, bleeding index (BI), suppuration (0/1), and probing depth (PD). PLI, suppuration, and PD will be evaluated at four sites per implant (mesiobuccal, buccal, distobuccal, and lingual/palatal) [20]. The PD will be measured to the nearest millimeter using a plastic graded probe (Hu-Friedy Mfg. Co., LLC, Chicago, IL, USA). The BI will be recorded at two sites per implant (buccal and lingual).

The PLI will be assessed as follows: 0, no plaque in the gingival margin area; 1, a thin layer of plaque on the tooth surface of the gingival margin area, but not visible on inspection if the side of the probe tip is used to scrape the plaque; 2, medium amount of plaque on the adjacent surface; or 3, a large amount of soft dirt in the gingival sulcus or the gingival margin area and the adjacent surface.

The BI will be assessed as follows: 0, no inflammation or bleeding on probing; 1, mild marginal inflammation (slight change in color or texture of any portion, but not the entire marginal or papillary gingival unit) and absence of bleeding on probing; 2, mild marginal inflammation (criteria as above but involving the entire marginal or papillary gingival unit) and slight gingival sulcus bleeding on probing; 3, moderate inflammation (glazing, redness, edema, and/or hypertrophy of the marginal or papillary gingival unit) and linear gingival sulcus bleeding on probing; or 4, severe inflammation (marked redness, edema, and/or hypertrophy of the marginal or papillary gingival unit) and bleeding on probing over the sulcus. When recording bleeding, the probing force will be no more than 15 g.

Two examiners will be trained prior to and during the trial to achieve maximum reproducibility of the measurements [21]. For continuous periodontal clinical parameters (PD), the standard error of the measurement will be evaluated. The average level of agreement between the two examiners will be considered satisfactory when greater than 90% (kappa test) for the other clinical variables.

The index developed by Mendez Caramês et al. [22] will be used to evaluate mechanical complications. If no alterations are present, an “alpha” classification will be attributed, and if minor chipping occurs (not requiring any intervention besides polishing or recontouring without the need for prosthesis retrieval), the prosthesis will be recorded as “bravo”; a “Charlie” classification will be attributed to the occurrence of major chipping, need for prosthesis retrieval, and laboratory intervention; and finally, a “delta” classification will indicate a fracture of the framework.

### X-ray assessment

Marginal bone loss (MBL): immediately after placement of the final prosthesis, a periapical radiograph of each implant will be obtained as well as every year afterward. For standardization, a paralleling technique will be conducted using an intramural digital system (Digora Toto, Soredex, Tuusula, Finland). Kodak Dental Imaging 6.1 software (Carestream Health, Inc., Rochester, NY, USA) will be used for radiographic analysis. The crestal bone level will be measured as the vertical distance between 2 mm below the implant–abutment interface and the most crestal part of the alveolar bone [23, 24]. MBL will be measured mesially and distally for each implant. In each group, peri-implant MBL will be measured to the nearest millimeter.

A peri-implantitis lesion is defined as having PD of ≥ 5 mm with positive suppuration or BI of ≥ 1 and radiographic evidence of bone loss (> 2 mm) or according to consensus [25]. Peri-implant mucositis is defined as being positive for suppuration or BI of ≥ 1 with no radiographic evidence of bone loss. A healthy implant site is defined as a PD of ≤ 4 mm, BI = 0, and no radiographic evidence of bone loss.

The incidences of peri-implantitis and peri-implant mucositis will be determined at 1, 3, and 5 years after final restoration.

### Laboratory assessment

#### Microbiological monitoring

##### Sample collection

Sulcus sampling will be performed immediately before prosthetic treatment and every 6 months after implantation of the final prostheses. Before sampling, antimicrobial mouth wash should not be used within the past 48 h and the patient should not eat within the past 1 h. Prior to sampling, clinical sites will be isolated and dried. Then, any supragingival/supramucosal plaque and calculus will be carefully removed. Submucosal plaque around one single implant will be sampled by inserting 4 sterile paper points (no. 30) into the base of the sulcus or pocket for 20 s. The paper points will be placed in labeled Eppendorf tubes, frozen, and transported to our laboratory for DNA extraction. Four paper points around one single implant will be grouped for analysis.

##### Processing of microbiological samples

Bacterial identification and classification will be determined by sequencing of the V1 and V3 regions of the 16S rRNA gene and amplification by polymerase chain reaction (PCR), as well as library preparations, library quality inspections, and quantifications of DNA samples of the oral flora. The identified TAG sequences will be used for sample differentiation. Qualified libraries will be sequenced with the Hiseq 2500 high-throughput sequencing platform (Illumina, Inc., San Diego, CA, USA). Paired-end reads obtained by Hiseq/Miseq sequencing will be spliced into one sequence and the target sequence subjected to quality control filtering. The filtered sequence will be compared with a reference database, and the chimeric sequence will be removed to obtain the final optimized sequence. Operational taxonomic unit (OTU) cluster analysis and species classification annotations are based on optimized sequences, diversity index analysis is based on OTU clustering results, and species structure and difference analyses are based on taxonomic information. Beta diversity analysis, principal co-ordinate analysis (PCoA), and linear discriminant analysis (LDA) effect size analysis will be used to compare differences in microbial diversity and in significant microbial species between the two treatment groups.

#### Surface roughness assessment

The tests will be performed before prosthetic delivery and every 6 months after implantation of the final prostheses. For standardization, surface roughness measurements will be made at six points around each abutment (mesial buccal, buccal, distal buccal, distal lingual, lingual, and mesial lingual). For each point, measurements and analysis will be repeated twice. The reproducibility will be assessed by calculating the intraclass correlation coefficient with a confidence interval of 95%.

### Randomization, allocation, and blinding

The subjects will be randomly allocated to one of two groups according to the prosthetic materials: titanium framework with acrylic resin veneering (group 1) or zirconia framework with/without ceramic veneering (group 2). The allocation of patients will be randomized using computer-generated permuted block randomization with an allocation ratio of 1:1. Randomization will be performed by sealed envelopes that will be opened after final impressions are obtained.

Microbiota analysis will be blinded after assignment to the intervention groups. Each sample will have a number associated with an allocation sequence, as well as information pertaining to the dental position and acquisition time. The technician conducting the PCR analysis will be blinded to the source of the sample. Interim statistical analysis will be performed at 1 and 3 years after implantation of the final prostheses. Final statistical analysis will be conducted at the end of the trial with the analyst blinded to both patient allocation and the interim analysis results.

### Sample size

The sample size has been calculated with NCSS-PASS software. At 5 years, peri-implant bone loss in metal-resin/metal-ceramic IFCDPs was 0.9 ± 0.4 mm and peri-implant bone loss in ceramic IFCDPs was 0.6 ± 0.1 mm [26]. In the proposed study, the criterion for significance is set at *α* = 0.05 (type I error) and *β* = 0.10 (type II error). The analysis is two-tailed. Assuming a dropout rate of 20%, in order to determine if there is a difference in the degree of bone loss between the two groups, 22 cases per group and 44 cases in total will be required. A previous study included 20 edentulous subjects who received two mandibular implants [10]. The abutments were either titanium or zirconium dioxide (non-submerged implant placement, within-subject comparison, left-right randomization). After 3 months, mean absolute counts of *Porphyromonas gingivalis* of the titanium vs. zirconia abutment were 1,000,000 ± 0 vs. 64,000 ± 36,770, respectively. However, the difference between groups was not evaluated in this study. Mean absolute counts (mean) for *Prevotella intermedia* of the titanium vs. zirconia abutment were 600,088 ± 952,117 vs. 3,600,089 ± 804,935, although the difference between the two groups was not evaluated. In this study, the criterion for significance will be set at *α* = 0.05 (type I error) and *β* = 0.10 (type II error). The analysis will be two-tailed. Assuming a dropout rate at 20%, in order to determine if there is a difference in the amounts of *P*. *gingivalis* and *P. intermedia* between the two groups, three patients per group and six patients in total will be required.

In summary, the total sample size will be 44 (22 per group).

### Statistical analysis

All statistical analyses will be conducted using IBM SPSS Statistics for Mac, version 19.0. (IBM Corporation, Armonk, NY, USA).

#### Clinical monitoring and X-ray assessment

Continuous variables will be described as the mean ± standard deviation or median. Grade and qualitative data will be described as a percentage. Age and other basic information between groups will be compared with the independent *t* test. Gender, implant survival rates, peri-implantitis rates, and peri-implant mucositis rates between the test and control groups will be compared using the chi-square test. PD and X-ray indices between groups will be compared using the independent *t* test. The differences in PLI and BI between the two groups will be evaluated, as well as the differences in PLI and BI over a period of 10 follow-ups. A generalized estimating equation will be used to eliminate possible internal correlations between multiple implants of the same patient. A Cox regression model will be used to exclude the influences of the shapes of the intaglio surface, the distances between the inserted implants, and other cofounders affecting the primary and secondary outcome variables. A probability (*p*) value of < 0.05 will be considered statistically significant.

#### Surface roughness assessment

Surface roughness of the two groups will be compared using the independent *t* test. A *p* value of < 0.05 will be considered statistically significant.

#### Microbiological monitoring

The mean counts (× 10^5^) of individual bacterial species and the percentage of the total DNA probe will be calculated initially for each implant, then per subject and averaged across patients between groups. Periodontal pathogens include *P. gingivalis*, *Fusobacterium nucleatum* subspecies, and *P. intermedia*, among others. The proportions for the species will be distributed into the six complexes and an “other” group, as proposed by Socransky et al. [27]. Differences in microbiological parameters between groups will be identified using the Wilcoxon signed-rank test. Adjustments for multiple comparisons [28] will be performed when the bacterial species are evaluated simultaneously. The level of significance will be set at 5%.

Alpha and beta diversity analyses will be performed using Primer7 software and the QIIME2 microbiome bioinformatics platform [29, 30]. Alpha diversity, Shannon’s diversity index of both species number and distribution, Margalef’s index of numbers, and Pielou’s index of evenness of distribution [31–33] will be analyzed, and the significance of the differences between groups will be derived using the unpaired Student’s *t* test. Beta diversity analysis includes visualization of data at multiple taxonomic levels, with unweighted and weighted UniFrac distance metrics in order to generate PCoA plots [34]. Analysis of similarity (ANOSIM) will be performed to determine whether there are significant differences in microbial communities between groups. White’s non-parametric test will be applied to test for differences in the abundance of specific microbiota between the groups, with a false discovery rate cutoff of 0.005 with the use of STAMP [35].

### Dissemination of results

The results of the trial will be published in international peer-reviewed journals. A summary of the study results will also be submitted to ClinicalTrials.gov to allow general access.

### Interim analyses

Interim statistical analysis will be performed at 1 and 3 years after the placement of the final prostheses. The analyst will be blinded to the allocation of the patients and will submit the analysis results to the Data and Safety Monitoring Board (DSMB), which will announce an early close to the trial as long as the dropout implant rate exceeds 20%.

### Withdrawal and missing data processing

The patients will be informed at the beginning of the study of the right to withdraw at any time without providing a reason. Even in the event of a withdrawal, the patient will still receive treatment.

If a participant has taken an antibiotic in the last 3 months before a follow-up visit, submucosa samples will be collected, the results of clinical examinations (surface roughness) will be recorded, and X-ray examinations will be conducted. The data obtained from this follow-up will be discarded. However, we still include this participant in the following visits and do the regular periodontal maintenance. If there was no antibiotic use within 3 months prior to the next follow-ups, the data will be recorded.

Any patients who reported smoking during the follow-up period will be excluded from the study.

The possibility of loss to follow-up was considered and calculated as a part of the sample size estimation.

## Discussion

The aim of the current study is the effect of the properties of the prosthetic material on peri-implant microbiota diversity and abundance. The authors hypothesize that the corrosion and aging of framework may have a crucial role in the selection of bacteria that adhere to the pellicles on the prosthesis surface. Considering that biofilm formation and the microbiota might further influence the long-term condition of the peri-implant mucosa and bone loss, our data will widen current views of the etiology of peri-implant diseases, promote updated treatment strategies, and prevent peri-implant diseases.

Specifically, by controlling surface roughness and analyzing submucosal microbiota, our research targeted at edentulous patients with full-arch implants will provide scientific evidence of the differences in the material properties of zirconia and titanium in terms of the influence on biofilm formation. A previous study indicated that a roughness average of 0.2 μm was the threshold for maximum reduction of bacterial adhesion on abutment surfaces [36, 37], while the national standard of 0.025 μm was adopted for all prostheses in this study. A previous in vivo study found that there was no universal optimum roughness that can prevent adhesion of all bacterial species [38], and surface charge, surface energy, surface topography, and material stiffness all influence the bacterial response. Thus, a clinical index was used to determine which material is more susceptible to biofilm formation. To compare the pathogenicity of plaque between the two groups, analysis of oral flora is crucial. As a possible limitation, although the surfaces of the two frameworks were polished to the same roughness at the time of restoration, changes to the surface roughness of the two materials due to long-term use could not be prevented. On the one hand, the oral cavity is an aggressive environment. Material surfaces in the mouth are covered by the salivary pellicle (up to 1000 nm thick [39]), which can also alter the nanotopography of restorative materials [40], thereby greatly influencing surface roughness. Mechanical stimulus, temperature, and pH conditions can also favor additional bacterial adhesion to the prostheses and alter material surface characteristics. On the other hand, during the implementation of this trial, regular periodontal maintenance (ultrasonic devices with polyetheretherketone-coated tips) combined with erythritol air-polishing will be conducted. The procedure will be performed by experienced dental hygienists in order to limit the effect of periodontal maintenance on the surface properties of dental zirconia ceramics and the effect of titanium or zirconia implant abutments on epithelial attachments after periodontal maintenance. Furthermore, the mechanical properties of zirconia frameworks with ceramic veneering differ from those of titanium frameworks veneered with acrylic resin. Previous studies have revealed that technical complications occasionally occur, although at low incidences [41, 42]. The incidences of wear and fracture are 19.4% and 0.7%, respectively, for titanium frameworks and 7.3% and 5.9% for zirconia [43]. In consideration of possible changes in roughness due to chipping, further analysis, including the incidence of chipping, is needed.

The results of this trial will support a tangible decision-making process for choosing appropriate dental implant-based prostheses for edentulous patients.

### Trial status

The trial is registered at Clinicaltrials.org, and the study is open for recruitment. The recruitment of the participants was initiated in May 2020 and will be completed in May 2022 (study protocol version: 2nd edition, 11 March 2020).

## Supplementary Information

**Additional file 1.** Protocol Amendments Record

**Additional file 2.** Trials structured Study Protocol template

**Additional file 3.** Consent form

**Additional file 4.** Study prorocol

## Data Availability

The authors declare that the study protocol was registered in February 2020 at ClinicalTrials.gov (registration no. ChiCTR2000029470). The data from the current study will be available on the ClinicalTrials.gov web site at: http://www.chictr.org.cn/searchproj.aspx?title=&officialname=&subjectid=&secondaryid=&applier=&studyleader=&ethicalcommitteesanction=&sponsor=&studyailment=&studyailmentcode=&studytype=0&studystage=0&studydesign=0&minstudyexecutetime=&maxstudyexecutetime=&recruitmentstatus=0&gender=0&agreetosign=&secsponsor=&regno=2000029470&regstatus=0&country=&province=&city=&institution=&institutionlevel=&measure=&intercode=&sourceofspends=&createyear=0&isuploadrf=&whetherpublic=&btngo=btn&verifycode=&page=1.

## Abbreviations

ANOSIM: Analysis of similarity
BI: Bleeding index
CAL: Clinical attachment level
DSMB: Data and Safety Monitoring Board
EP: Eppendorf
IFCDPs: Implant-supported fixed complete dental prostheses
LDA: Linear discriminant analysis
MBL: Marginal bone loss
OHI: Oral hygiene instruction
OTU: Operational taxonomic unit
PCoA: Principal co-ordinate analysis
PCR: Polymerase chain reaction
PD: Probing depth
RCT: Randomized clinical trial
SPIRIT: Standard Protocol Items: Recommendations for Interventional Studies
