# Supplementary material for: Comparison of peri-implant submucosal microbiota in arches with zirconia or titanium implant-supported fixed complete dental prostheses: a study protocol for a randomized controlled trial
Source: Trials. 2020 Nov 27;21:979. doi: 10.1186/s13063-020-04853-7 (PMC7694361; doi:10.1186/s13063-020-04853-7)
Supplement: Supplementary file 2 — Additional file 2. Trials structured Study Protocol template [file 13063_2020_4853_MOESM2_ESM.docx]

**Protocol Amendments Record**

| No. | Ver. | Amendment Date | Reviser/Composer | Sections Being Amended |
| --- | --- | --- | --- | --- |
| 1 | 1 | 2020-02-24 | Zhaoguo Yue, Jingwen Yang and Jianxia Hou | Draft Composition |
| 2 | 2 | 2020-03-11 | Zhaoguo Yue and Jianxia Hou | Primary parameters and Challenges  (Final Ver.) |
| 3 |  |  |  |  |
| 4 |  |  |  |  |
| 5 |  |  |  |  |
| 6 |  |  |  |  |
