# Supplementary material for: Comparison of peri-implant submucosal microbiota in arches with zirconia or titanium implant-supported fixed complete dental prostheses: a study protocol for a randomized controlled trial
Source: Trials. 2020 Nov 27;21:979. doi: 10.1186/s13063-020-04853-7 (PMC7694361; doi:10.1186/s13063-020-04853-7)
Supplement: Supplementary file 3 — Additional file 3. Consent form [file 13063_2020_4853_MOESM3_ESM.docx]

***Trials* structured Study Protocol template**

**Administrative information**

**Author details {5a}**

Jing wen Yang, Zhongning Liu, Lin Tang & Jianzhang Liu: Department of Prosthodontics, Peking University School and Hospital of Stomatology; National Engineering Laboratory for Digital and Material Technology of Stomatology; Research Center of Engineering and Technology for Digital Dentistry of Ministry of Health; Beijing Key Laboratory of Digital Stomatology, Beijing, PR China;

Pingyi Jia: Department of the Fourth Clinical Division, Peking University School and Hospital of Stomatology; National Engineering Laboratory for Digital and Material Technology of Stomatology; Research Center of Engineering and Technology for Digital Dentistry of Ministry of Health; Beijing Key Laboratory of Digital Stomatology, Beijing, PR China;

Qi Liu: BYBO Dental Hospital, Qinian Street, Dongcheng District, Beijing 100062 P.R.China;

Jiaxia Hou, Churen Zhang & Zhaoguo Yue Department of Periodontology, Peking University School and Hospital of Stomatology; National Engineering Laboratory for Digital and Material Technology of Stomatology; Research Center of Engineering and Technology for Digital Dentistry of Ministry of Health; Beijing Key Laboratory of Digital Stomatology, Beijing, PR China

**Name and contact information for the trial sponsor {5b}**

Jing wen yang

Address: No. 22, Zhongguancun Avenue South, Haidian District, Beijing 100081, PR China.

Email: jingwen.yang@foxmail.com

Tel.: +86 15101157982

**Role of sponsor {5c}**

The clinical study is being conducted thanks to the contribution of Jingwen Yang: she designed the trial and she will be the general study coordinator. She also participated in the development of this manuscript together with Pingyi Jia. Jianzhang Liu is in charge of the recruitment of the participants. Zhaoguo Yue is responsible for clinical evaluation, microbiota sample collection and maintenance of the participants. Chunren Zhang will do microbiological samples processing and analysis. Qi Liu will perform X-ray measurement and do interim statistic analysis at year 1 and year 3. Qi Liu will be blinded to the allocation of the patients. He will submit the analysis results to Jiaxia Hou. Jiaxia Hou, together with other specials: Zhongning Liu and Lin Tang, will consist data and safety monitoring board (DSMB). DSMB will communicate with Jingwen Yang as long as DSMB group found this trial could be early terminated. Pingyi Jia will perform X-ray measurement and do final statistic analysis at the end of this trial, and she will be blinded to both the allocation of the patients and to the interim analysis results from Qi Liu. She also participated in the development of this manuscript. Pingyi Jia and Jingwen Yang contribute equally to this article. Other authors were invited to comment on the paper and approved the final draft.

**Composition of the coordinating centre and trial steering committee {5d}**

Recording of clinical indicators will be done jointly by two regular researchers. The two researchers will check with each other during the collection process (the results must be approved in pairs to record). Gingival crevicular fluid samples will be collected by two researchers with standardized training. Zhongning Liu and Chunren Zhang will responsible for double data entry. A research Supervisor: Haidong Zhang, will be employed to audit trial conduct per month. He will be independent from investigators and the sponsor, but he has channels to communicate with sponsor. His job includes check the enrollment of researchers, the completion of follow-up, and the filling of forms regularly.

A research Supervisor: Haidong Zhang, will be employed to audit trial conduct per month. He will be independent from investigators and the sponsor, but he has channels to communicate with sponsor. His job includes check the enrollment of researchers, the completion of follow-up, and the filling of forms regularly. He is responsible for data monitoring.

**Methods: Participants, interventions and outcomes**

**Explanation for the choice of comparators {6b}**

Considered outcomes include: implant survival rate, peri-implant plaque index, peri-implant mucosal tissue conditions, marginal bone resorption, peri-implant submucosal bacteria species, mechanical complicate rate, surface roughness.

Clinical signs of peri-implant mucosal inflammation may include bleeding on probing, erythema, swelling, and suppuration. BI and suppuration are two common comparators.

A cause‐effect relationship between experimental accumulation of oral biofilms around dental implants and the development of experimental peri‐implant mucositis has been demonstrated in humans. In this study, we chose plaque index and microbiota as comparators.

Peri-implantitis is characterized by inflammation in peri-implant tissues and loss of supporting bone. X-ray measurement of marginal bone loss is a very important comparator to diagnose peri-implantitis.

To evaluate technique complications, chipping, fracture, lossening of screws, and decementation are recorded. That is because all these complications will provide favorable condition for bacterial colonization and reproduction. And for the same reason, surface roughness of the prosthesis need to be tested.

**Intervention description {11a}**


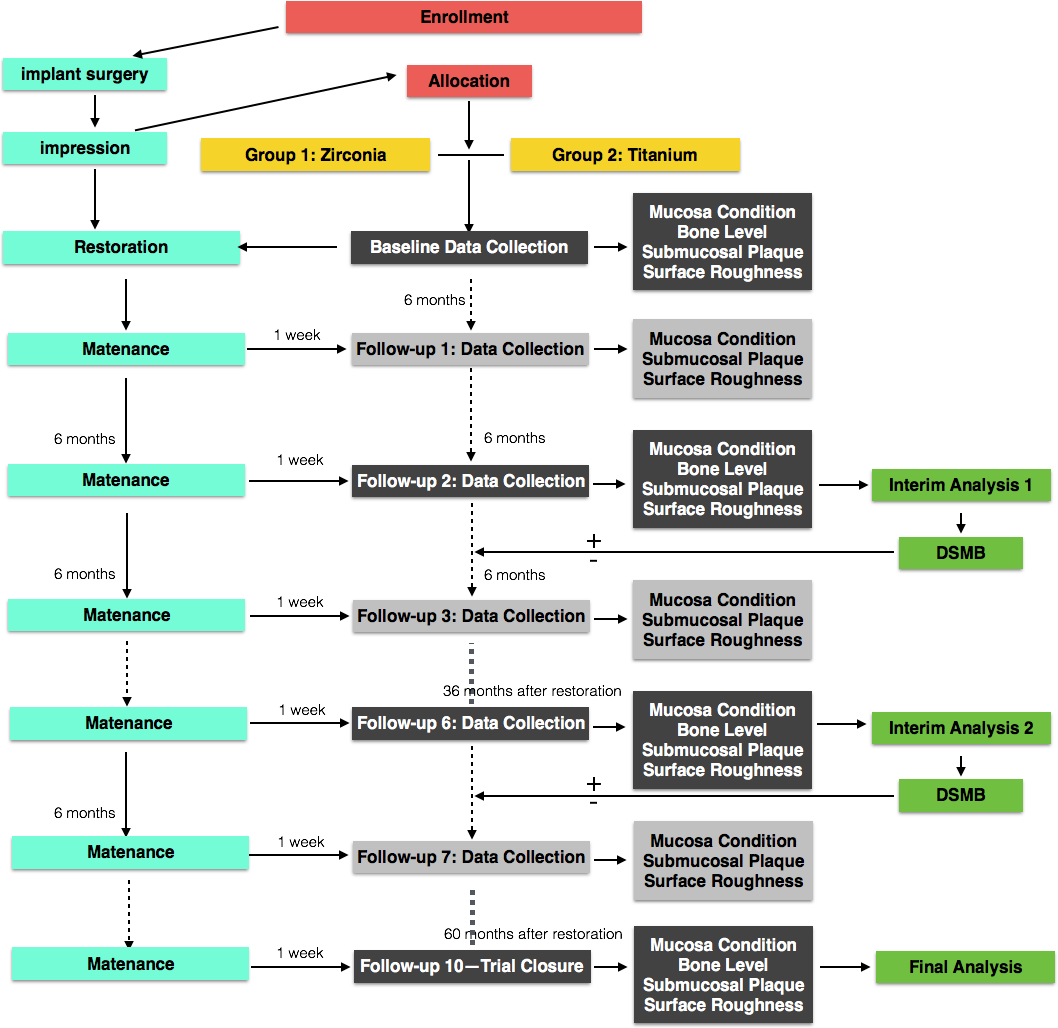


**Criteria for discontinuing or modifying allocated interventions {11b}**

Patients will be informed that they have the right to withdraw from the study at any time without giving reasons. Regardless of withdrawal, patients will be provided with any treatment requested by them. The results of the study will be published in international peer-reviewed journals. A summary of the study results will also be saved at Clinicaltrials.gov to allow general access to obtain findings.

Withdrawal mechanism: 1. Patients whose systemic conditions do not meet the inclusion criteria; 2. Patients who cannot continue to use the restorations due to mechanical or biological complications, such as fracture of framework and loose of implants. 3. Patients who cannot continue to follow up. 4. Patients who use antibiotics within 3 months before visit, will be excluded from microbiota sampling collection for this singling visit.

**Strategies to improve adherence to interventions {11c}**

The trial will be lasted for five years, and the difference between two groups will be analyzed at year 1, year 3 and year 5. We will preform regular periodontal maintenance to these patients, including supra-gingival ultrasonic debridement, air-polishing, oral hygiene instrument. Telephone interviews will be performed every 3 months, in order to monitor the implementation of oral hygiene measures, to collect the complication information, to give appropriate remote guidance and to make an appointment to review.

**Relevant concomitant care permitted or prohibited during the trial {11d}**

we will perform regular periodontal maintenance to these patients, including supra-gingival scaling, air-polishing, oral hygiene instrument. But anti-biotic drugs are not recommended . In each visit, before sampling, we will ask the patient about general conditions and drug uses in the last three months, including sub-gingival chlorhexidine irragation, oral antibiotic, etc. Then, clinical examination and X-ray examination will be conducted. If general conditions changes and drug uses achieve exclusion criteria, microbiota sampling will not be collected at this time point. If general conditions changes and drug uses do not achieve exclusion criteria, microbiota sample would be collected.

**Assignment of interventions: allocation**

**Implementation {16c}**

Jingwen Yang will generate the allocation sequence, Jianzhang Liu and Jingwen Yang will enroll participants, and Jingwen yang will assign participants to interventions.

**Assignment of interventions: Blinding**

**Who will be blinded {17a}**

Sample analysis of microbiota in laboratory will be blinded after assignment to interventions. Each sample has a number associated with an allocation sequence, dental position information, and acquisition time information. The analyst of the PCR laboratory does not know the source of the sample. Qi Liu will perform x-ray measurement and do interim statistic analysis 1 year and 3 years after final prostheses. Qi Liu will be blinded to the allocation of the patients. Pingyi Jia will perform x-ray measurement and do statistic analysis at the end of this trial, and she will be blinded to both the allocation of the patients and the interim analysis results from Qi Liu.

**Procedure for unblinding if needed {17b}**

None.

**Data collection and management**

**Plans for assessment and collection of outcomes {18a}**

Recording of clinical indicators will be done jointly by two regular researchers. The two researchers will check with each other during the collection process (the results must be approved in pairs to record). The two examiners were trained and calibrated prior to and during the trial, in order to achieve maximum reproducibility in the measurements. The methodology used for the inter-examiner and intra- examiner calibration was recommended by Araujo et al. [3], where the standard error of measurement for continuous periodontal clinical parameters (PD and CAL) is evaluated. For the other clinical variables, the average level of agreement between the examiners is determined and considered satisfactory when greater than 90% (Kappa test). Gingival crevicular fluid samples will be collected by two researchers with standardized training. The sampling operation will be performed in full accordance with the protocol.

**Plans to promote participant retention and complete follow-up {18b}**

During the implementation phase of the experiment, the subject's compliance should be improved as much as possible. For example, explain the details of the experiment to the subject so that he has a sufficient understanding of the risks, benefits, and the content that needs to be cooperated. And improve the quality of medical care, provide convenient measures, establish a good doctor-patient relationship, reduce unnecessary examinations and auxiliary treatment.

Missed data are not included in the final data analysis, only data analysis before the missed time node. Sample size calculation was performed accounting for possible loss to follow-up.

**Data management {19}**

Recording of clinical indicators will be done jointly by two regular researchers. The two researchers will check with each other during the collection process (the results must be approved in pairs to record). Gingival crevicular fluid samples will be collected by two researchers with standardized training. Zhongning Liu and Chunren Zhang will responsible for double data entry. A research Supervisor: Haidong Zhang, will be employed to audit trial conduct per month. He will be independent from investigators and the sponsor, but he has channels to communicate with sponsor. His job includes check the enrollment of researchers, the completion of follow-up, and the filling of forms regularly.

**Statistical methods**

**Methods for additional analyses (e.g. subgroup analyses) {20b}**

Within the group, the flora analysis will be performed according to the implantation site: the difference between the anterior and posterior teeth, the difference between the upper and lower jaw, the difference between the upright implant and the inclined implant, and the difference between the inflammation site and the non-inflammatory site.

**Methods in analysis to handle protocol non-adherence and any statistical methods to handle missing data {20c}**

Sample size calculation are performed accounting for possible loss to follow-up. Moreover, we will account for the data not missing at random due to unbalanced loss to follow-up by handling drop-outs as nonsuccess or nonsurvival using the intention-to-treat principle. If patients are lost to follow-up, the reason for not completing follow-up should be recorded..

**Interim analyses {21a}**

Qi Liu will perform X-ray measurement and do interim statistic analysis 1 year and 3 years after final prostheses. Qi Liu will be blinded to the allocation of the patients. He will submit the analysis results to Jiaxia Hou regularly. Jiaxia Hou, together with other specials, will consist data and safety monitoring board DSMB. DSMB will announce an early close as long as DSMB find the drop-out implants or patients exceed 20% of the enrolled implants or patients.

**Oversight and monitoring**

**Composition of the data monitoring committee, its role and reporting structure {21a}**

Haidong Zhang will be employed for data monitoring, if he finds incomplete data entry, he will communicated with Jingwen Yang in 24 hours.

**Adverse event reporting and harms {22}**

The complications of implant treatment will be recorded during each semi-annual follow-up. The records will be published if any adverse event happens.

**Frequency and plans for auditing trial conduct {23}**

A research Supervisor: Haidong Zhang, will be employed to audit trial conduct per month. He will be independent from investigators and the sponsor, but he has channels to communicate with sponsor. His job includes check the enrollment of researchers, the completion of follow-up, and the filling of forms regularly.

**Plans for communicating important protocol amendments to relevant parties (e.g. trial participants, ethical committees) {25}**

The sponsor will communicate with ethical committees when major adjustments are planned to be made for the research protocol.

**Who will take informed consent? {26a}**

Jianzhang Liu and Jingwen Yang are responsible for the recruitment of the participants and signing inform consents with the participants in the Peking University School and Hospital of Stomatology center.

The systematic health condition of all the participants will be recorded and all the participants should fulfill the inclusion criteria. Before the implant treatment, all patients will receive clinical and radiographic assessment. All patients will be required to receive oral hygiene instructions from Zhaoguo Yue before implant surgery.

**Additional consent provisions for collection and use of participant data and biological specimens {26b}**

None

**Confidentiality {27}**

Participation in the personal data and specimen information are confidential. Names, ID numbers, addresses, telephone numbers, or any directly identifiable information in research records will not be leaked outside Peking University Stomatological Hospital. For research information transmitted outside Peking University Stomatological Hospital, we will use a unique number to represent it. The coded information will be properly stored in Peking University Stomatological Hospital.

Participants status information will not be disclosed to members outside the research team unless permission is obtained from themselves. At the end of the study, the data will be destroyed. All research members and research sponsors are required to keep volunteers confidential. To ensure that the research is conducted in accordance with regulations, members of government management departments or ethics review committees can consult the personal data of volunteers at the research unit as required.

Volunteers can request access to their personal information (such as name and address) at any time, and can modify this information if necessary.

**Access to data {29}**

Pingyi Jia will perform X-ray measurement and do final statistic analysis at the end of this trial, and she will be blinded to both the allocation of the patients and to the interim analysis results from Qi Liu.

**Provisions for post-trial care {30}**

The two interventions in this study are routine clinical treatments with stable and reliable efficacy, which will not cause possible harm to patients. The data collection do not increase patients’ visits, treatment costs and time.

**Dissemination plans {31a}**

The results of the study will be published in international peer-reviewed journals. A summary of the study results will also be saved at Clinicaltrials.gov to allow general access to obtain findings.

**Plans to give access to the full protocol, participant level-data and statistical code {31c}**

Not applicable.
