# Supplementary material for: Comparison of peri-implant submucosal microbiota in arches with zirconia or titanium implant-supported fixed complete dental prostheses: a study protocol for a randomized controlled trial
Source: Trials. 2020 Nov 27;21:979. doi: 10.1186/s13063-020-04853-7 (PMC7694361; doi:10.1186/s13063-020-04853-7)
Supplement: Supplementary file 4 — Additional file 4. Study prorocol [file 13063_2020_4853_MOESM4_ESM.docx]

**知情同意书**

**尊敬的患者：**

您将被邀请参加一项由北京大学口腔医院修复科杨静文医生主持的研究。这是一项**探究全牙列种植修复材料与口腔菌群相关性的研究。**本研究预计将有20名受试者自愿参加。请您仔细阅读本知情同意书并慎重做出是否参加研究的决定。当您的研究医生或者研究人员和您讨论知情同意书的时候，您可以让他/她给您解释您看不明白的地方。我们鼓励您在做出参与此项研究的决定之前，和您的家人及朋友进行充分讨论。若您正在参加别的研究，请告知您的研究医生或者研究人员。本研究已经得到北京大学口腔医院生物医学伦理委员会的审查和批准。本研究的内容/性质、风险、不便或不适及其他重要信息如下：

**背景和目的**

随着无牙颌种植修复的治疗效果获得学界肯定，远期成功率逐渐受到研究者们关注。生物学并发症是影响远期成功率的重要因素。除了与患者自身的口腔卫生维护能力、口腔固有菌群相关，种植体周围的菌群种类还与种植修复材料的生物相容性、表面粗糙度相关，并由此影响种植体周围的组织的生理状态。以钛合金为主体的上部修复体在多年的临床使用中表现出了良好的生物相容性和机械性能。但金属氧化带来的局部酸碱度改变以及金属表面电荷对菌群粘附的影响不容忽视。氧化锆作为新兴的支架材料，具有良好的生物相容性、机械性能和抗氧化腐蚀的特点，但加工成本略高。本研究旨在探讨不同种植上部修复材料的无牙颌种植修复患者的口腔龈下菌群特点，以及其与生物并发症的相关性。为上部修复材料的选择提供科学依据。

**研究过程/方法：**


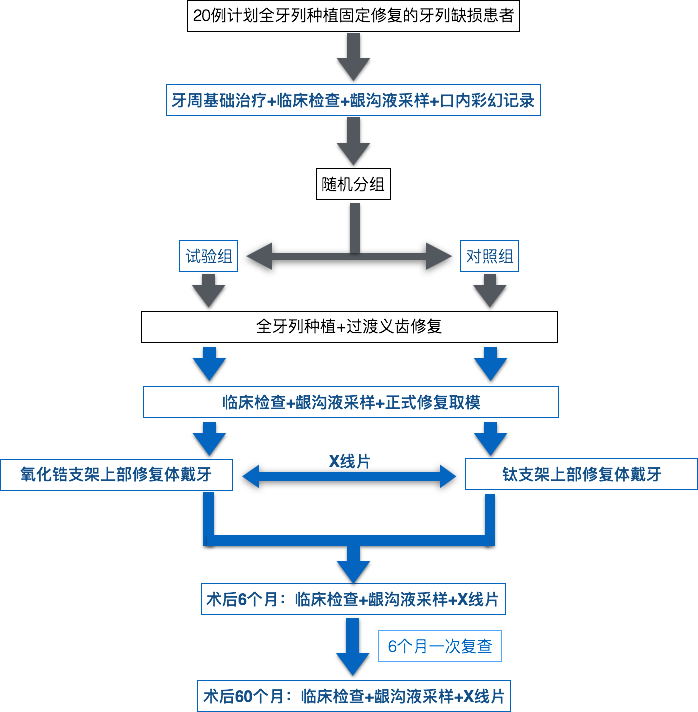


**研究信息的获取**

您有权获知研究信息；研究结果通过论文等方式发表，但是关于您的研究信息，我们会用一个独一无二的编号代表您。编码信息将被妥善存放在北京大学口腔医院，不对外公开。

**您需履行的责任**

本研究将持续5年半,10次随访（种植修复后每6个月一次随访）。在此期间，您需要按上述流程图依照计划时间点复诊——种植治疗完成后每半年需要复查一次，进行口腔卫生检查、种植体临床检查并留取影像学资料和龈沟液。龈沟液的获取为无创操作，在口腔检查的同期用试纸采集。除此以外的检查操作都是常规复查项目。在随访期间，您还需要保持良好的口腔卫生。以上不增加您的就诊次数、治疗费用和时间。

**参加该研究对您生活的影响**

1. 参与本试验的受试者，需要按照医生的要求定期复查
2. 女性患者需要避免怀孕

当您决定是否参加本研究时，请仔细考虑如上所列的检查和随访对您的日常工作、家庭生活等的影响。考虑每次回访的时间与交通问题。若您对试验涉及的检查和步骤有任何疑问，可以向医生咨询。

**参加此研究的风险和不良反应**

研究过程中您可能会面临种植手术术中及术后常见的风险及不良反应（详见种植手术知情同意书），除此以外并无特殊的风险和不良反应。

研究过程中我们会对种植修复完成后可能出现的常见并发症进行监测、记录和及时的处理，包括：人工牙脱落、崩瓷、基托折裂、螺丝折断、基台折裂、种植体折裂等。

研究过程中拍摄的CBCT以及根尖片放射剂量很低，不会对身体健康产生不良影响，也是种植修复后的常规检查方法。我们会监测所有研究对象的任何不良反应。如果您在访视之间出现任何不良反应，请及时给您的研究医生打电话咨询。

您需告诉您的家人或与您亲近的朋友您正在参加一项临床研究，他们可以注意上面描述的事件。如果他们对您参加研究有疑问，您可以告诉他们怎样联系您的研究医生。

**是否有其他的治疗选择**

您可以选择不参加本项研究，这对您获得常规治疗不会带来任何不良影响。

**随机分组的方法**

参与本研究获得编号后，受试者会被随机分至组1和组2（分别对应氧化锆支架上部修复体和钛支架上部修复体）。在手术治疗前，受试者和医生都无法获知实际的分组情况，双方也无权进行选择和更改。

**参加本研究会给予的费用、补偿及报酬**

无。

本研究会为您妥善安排种植手术。随访期间受试者所需相关治疗将获得牙周、修复或种植专家的诊治。

在临床试验中可能出现的事件是修复螺丝遗失，如若出现项目负责人会免费配备新的螺丝，保证修复体的正常使用。

对于您同时合并的其他疾病所需的治疗和检查，将不在免费的范围之内。

与本研究无关的种植修复后常见并发症处理的治疗费用，不在免费范围之内。

**您个人信息的保密**

如果您决定参加本项研究，您参加试验及在试验中的个人资料和标本资料均属保密。研究记录中您的姓名、身份证号码、地址、电话、或者任何可以直接辨别您身份的信息不会被泄露到北京大学口腔医院之外。对那些传送到北京大学口腔医院之外的关于您的研究信息，我们会用一个独一无二的编号代表您。编码信息将被妥善存放在北京大学口腔医院。

可以识别您身份的信息将不会透露给研究小组以外的成员，除非获得您的许可。研究结束时，资料将被销毁。所有的研究成员和研究申办方都被要求对您的身份保密。为确保研究按照规定进行，必要时，政府管理部门或伦理审查委员会的成员按规定可以在研究单位查阅您的个人资料。我们将在法律允许的范围内，尽一切努力保护您个人医疗资料的隐私。

在任何时候，您可以要求查阅您的个人信息（比如您的姓名和地址），如有需要可以修改这些信息。

**自由退出**

**参加本研究是完全自愿的，**您可以拒绝参加研究，或者在研究过程中的任何时候选择退出研究，该决定不会影响您接受种植修复治疗。如果您不参加本研究，或中途退出研究，您的资料和标本将被销毁。

如果您决定退出本研究，请提前通知您的研究医生。

**联系方式**

如果您有与本研究有关的问题，或您在研究过程中发生了任何不适，或有关于本项研究参加者权益方面的问题，您可以与杨静文医师联系，办公电话：010-82195991；手机：15101157982；或与北京大学口腔医院生物医学伦理委员会联系，办公电话：010-82195759。

**试验后利益分享**

无

**知情同意签署页**

**受试者或监护人同意声明：**

我已经阅读了上述有关本研究的介绍，对参加本研究可能产生的风险和受益充分了解。我是自愿同意参加本文所介绍的临床研究。

受试者正楷姓名：受试者签名：

签名日期： 手机号：

监护人/代理人正楷姓名（如有）： 监护人/代理人签名：

监护人/代理人与患儿的关系：

签名日期：手机号：

**研究者声明：**

我确认已向患者解释了本研究的详细情况，特别是参加本研究可能产生的风险和收益。

研究者正楷姓名： 研究者签名：

签名日期： 手机号：
